# Heart Failure Incidence and Risk Factors in U.S. Adults Receiving Bezlotoxumab: A Large Database Analysis

**DOI:** 10.3390/idr18020028

**Published:** 2026-03-31

**Authors:** Chia-Yu Chiu, Daniel B. Chastain, Joseph Sassine, Andrés F. Henao-Martínez

**Affiliations:** 1 Division of Infectious Diseases, Department of Medicine, University of Colorado, Aurora, CO 80045, USA; andres.henaomartinez@cuanschutz.edu; 2 Department of Clinical and Administrative Pharmacy, University of Georgia College of Pharmacy, Albany, GA 31701, USA; daniel.chastain@uga.edu; 3 Infectious Diseases Section, Department of Medicine, University of Oklahoma Health Sciences Center, Oklahoma City, OK 73104, USA; joseph-sassine@ouhsc.edu

**Keywords:** bezlotoxumab, *C. difficile* toxin B, heart failure exacerbations, *Clostridium difficile* infection, adverse event

## Abstract

Background: Bezlotoxumab is used to prevent recurrent *Clostridioides difficile* infection. Although well tolerated, heart failure (HF) exacerbations have been reported as adverse events in clinical trials. This study evaluates the incidence and predictors of HF exacerbation following bezlotoxumab. Methods: We used the TriNetX research database to identify U.S. adults who received bezlotoxumab and stratified them into three groups based on HF history: no HF, HF with preserved ejection fraction (HFpEF), and HF with reduced ejection fraction (HFrEF). The 90-day cumulative incidence of HF events and mortality were assessed. Cox proportional hazard models identified predictors of HF events. Results: Among 2515 patients, 89% had no HF history, 4% had HFpEF, and 7% had HFrEF. The 90-day HF event rates were 1%, 29%, and 52% for the no HF, HFpEF, and HFrEF groups, respectively (*p* < 0.001). The 90-day all-cause mortality was 0.9%. Corresponding 90-day all-cause mortality rates were 0.04%, 4%, and 11%, respectively (*p* < 0.001). Independent positive predictors of HF events included HFrEF (aHR 19.400), HFpEF (adjusted hazard ratio [aHR] 8.632), heart transplant (aHR 7.485), hyperlipidemia (aHR 3.184), valvular heart disease (aHR 2.267), chronic kidney disease stage ≥ 3 (aHR 1.715), and ischemic heart disease (aHR 1.987). Protective factors included non-cardiac solid organ transplant (aHR 0.333). Conclusions: Bezlotoxumab appears safe in patients without HF history but is associated with a significantly increased risk of HF exacerbation in those with pre-existing HF, especially HFrEF.

## 1. Introduction

Bezlotoxumab, a monoclonal antibody targeting *Clostridioides difficile* toxin B, was approved by the U.S. Food and Drug Administration (FDA) in 2016 for recurrent *C. difficile* infection (CDI) prevention [1]. In MODIFY I/II trials, an increased incidence of heart failure (HF) exacerbations was observed among participants with a history of HF who received bezlotoxumab (13%), compared to the placebo (5%) [1]. Mortality was 19% in the bezlotoxumab versus 13% in the placebo, although the causes of death were heterogeneous [1]. Importantly, HF exacerbations and deaths were not infusion-related but occurred throughout the 12-week follow-up period [1]. Based on these findings, the FDA issued a warning stating that “in patients with a history of congestive heart failure, bezlotoxumab should be reserved for use when the benefit outweighs the risk” [1]. On the other hand, a few studies also examine whether bezlotoxumab can be used after the first episode of CDI [2]. However, real-world data describing the incidence of HF events following bezlotoxumab administration remain scarce. Therefore, we used the TriNetX database to examine HF events in CDI patients who received bezlotoxumab.

## 2. Materials and Methods

### 2.1. Study Design

We identified adult (age ≥ 18) CDI patients who received bezlotoxumab from U.S. healthcare institutions within the TriNetX database between November 2015 and December 2025 [3]. ICD-10 codes were used to identify CDI diagnoses, relevant medical history, and HF exacerbations. RxNorm codes were used to identify patients who received bezlotoxumab and other medications of interest. The supplemental methods and Tables S1–S3 provide the study design. The index date was defined as the date of bezlotoxumab administration. The event was defined as the date of HF exacerbation. Our group has published studies utilizing this database to evaluate drug safety [4,5].

### 2.2. Definition

Patients were followed for 90 days after bezlotoxumab infusion to evaluate HF events and mortality, consistent with the follow-up period used in clinical trials [1]. Patients were stratified into three groups: (1) without HF history; (2) history of HF preserved ejection fraction (HFpEF); (3) history of HF reduced ejection fraction (HFrEF). Heart failure event was defined as (1) identified HF (ICD-10 I50) in patients without HF history after receiving bezlotoxumab, (2) identify HF exacerbation (ICD-10 I50.2×, I50.3×, I50.4×) in patients with history of HFpEF or HFrEF after receiving bezlotoxumab.

### 2.3. Outcome Measures

The primary outcomes were (1) the 90-day cumulative incidence of HF events, (2) the 90-day all-cause mortality after bezlotoxumab administration, stratified by HF history (no HF history, HFpEF, HFrEF). The secondary outcome was to identify independent predictors associated with HF events.

### 2.4. Statistical Analysis

Descriptive statistics were reported as median (interquartile range [IQR]) for continuous variables and numbers (percentages) for categorical variables. Continuous variables were compared using the Kruskal–Wallis H test. The cumulative incidence of HF and probability of survival were estimated using the Kaplan–Meier survival analysis, with between-group differences assessed by the log-rank test. Multivariable Cox proportional hazards models were used to evaluate associations with HF events. Statistical analyses were conducted using the software SAS version 9.4 (SAS Institute Inc., Cary, NC, USA). The figure was produced using GraphPad Prism version 10.5.1. (GraphPad Software, San Diego, CA, USA).

## 3. Results

**Figure 1:**
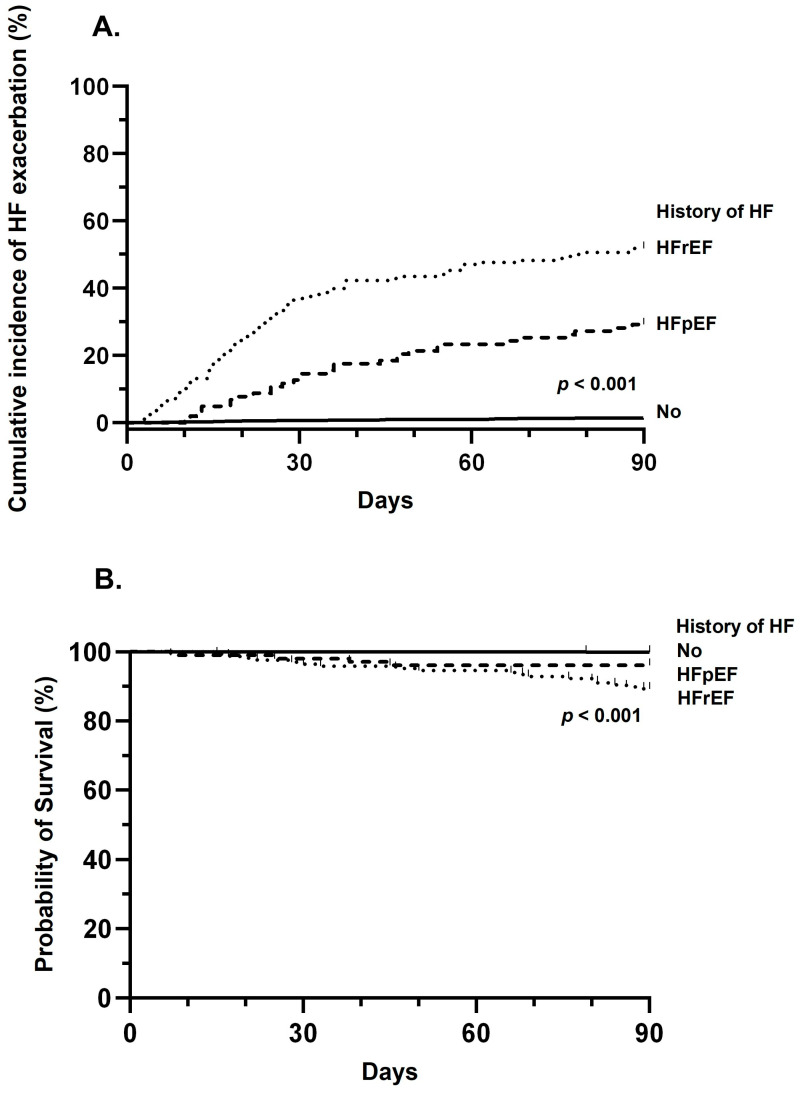
(**A**) 90-day cumulative incidence curves of heart failure exacerbation. (**B**) 90-day Kaplan–Meier survival curves for mortality. Both were stratified by the history of heart failure. Comparisons between groups were made using the log-rank test (*p* < 0.001). Abbreviations: HFpEF, heart failure with preserved ejection fraction; HFrEF, heart failure with reduced ejection fraction.

We identified 2515 individuals who received bezlotoxumab, including 2244 (89%) without HF history, 103 (4%) with a history of HFpEF, and 168 (7%) with a history of HFrEF (Figure S1). The 90-day cumulative incidence of HF events was 6%. When stratified by HF history, the 90-day incidence was 1% in patients without HF, 29% in those with HFpEF, and 52% in those with HFrEF (log-rank test, *p* < 0.001) (Figure 1A). The median time from bezlotoxumab infusion to HF event was 29 days (IQR 16–64) in patients without HF, 33 days (IQR 20–53) in those with HFpEF, and 21 days (13–35) in those with HFrEF (*p* = 0.009).

The 90-day all-cause mortality of the study population was 0.9%. The 90-day all-cause mortality was 0.04% in patients without HF history, 4% in those with HFpEF, and 11% in those with HFrEF (log-rank test, *p* < 0.001) (Figure 1B). Compared to patients without HF, the hazard ratio (HR) for mortality was 89.6 (95% confidence interval [CI], 11.1–722.6) for patients with HFpEF, and 252.4 (95% CI, 47.4–1345.7) for those with HFrEF.

The final multivariable Cox regression model identified seven independent predictors of HF events following bezlotoxumab: history of HFpEF (aHR, 8.632; 95% CI, 4.497–16.568; *p* < 0.001), history of HFrEF (aHR, 19.400; 95% CI, 10.757–34.988; *p* < 0.001), chronic kidney disease (CKD) stage ≥ 3 (aHR, 1.715; 95% CI, 1.029–2.857; *p* = 0.039), hyperlipidemia (aHR, 3.184; 95% CI, 1.793–5.655; *p* < 0.001), history of heart transplantation (aHR, 7.485; 95% CI, 1.731–32.306; *p* = 0.007), ischemic heart disease (aHR, 1.987; 95% CI, 1.128–3.502; *p* = 0.018), and valvular heart disease (aHR, 2.267; 95% CI, 1.373–3.742; *p* = 0.001) (Table 1). In contrast, non-cardiac solid organ transplantation (aHR, 0.333; 95% CI, 0.161–0.688; *p* = 0.003) was independently associated with a reduced risk of HF events.

## 4. Discussion

In this study, a prior history of HF, whether HFpEF or HFrEF, was the strongest predictor of HF events following bezlotoxumab infusion. Classic HF risk factors, including CKD, ischemic heart disease, and valvular heart disease, also independently increased risk. Among patients with solid organ transplants, heart transplantation was associated with higher HF risk (e.g., cardiac allograft vasculopathy or chronic graft dysfunction), whereas non-cardiac solid organ transplantation was protective. Patients with a history of HFrEF experienced HF events sooner post-infusion, and had a higher all-cause mortality rate compared to those without HF or with HFpEF.

Outside of clinical trials, six retrospective studies have reported HF events after bezlotoxumab, though these were limited by small sample sizes and often lacked HF history data (Table 2) [6,7,8,9,10,11]. Treating physicians frequently attributed HF exacerbations to underlying comorbidities rather than to bezlotoxumab itself [6,8]. Our observed 1% incidence of HF events in patients without prior HF aligns with the 2% reported in clinical trials [1]. However, HF exacerbation rates were substantially higher in patients with HF history (29% in HFpEF, 52% in HFrEF). To date, only one small study (n = 41) differentiated HF subtypes, reporting no HF events in HFpEF and 13% in HFrEF [11]. In real-world practice, patients with known HF might be excluded from bezlotoxumab treatment due to safety concerns. Consequently, those who received bezlotoxumab likely represent a highly selected subset at increased risk of *C.difficile* recurrence, with greater comorbidity burden and poorer functional status. This selection bias may have led to high HF events and significant all-cause mortality following bezlotoxumab infusion in our study.

Cardiovascular adverse events are known with some monoclonal antibodies used in oncology due to cytokine modulation and off-target cardiomyocyte effects [12]. In contrast, to our knowledge, bezlotoxumab directly neutralizes *C. difficile* toxin B, with no in vitro evidence of direct cardiotoxicity. In this study, some, but not all, cardiovascular risk factors or comorbidities were identified as predictors of HF events. We postulate that volume overload (such as hydration during CDI treatment) in vulnerable individuals with pre-existing HF or cardiovascular comorbidities is the likely mechanism of HF exacerbation, rather than direct drug toxicity. Although bezlotoxumab is FDA-approved for use in patients with recurrent CDI, a few studies also showed it to be effective and safe in patients with a first episode of CDI [2]. Nevertheless, the incidence of HF events was not reported in this specific subgroup [2]. Unfortunately, bezlotoxumab was discontinued by the manufacturer in January 2025. Since HF increases the risk of mortality in CDI [13], future CDI prevention studies should prioritize cardiovascular safety monitoring, particularly in patients with underlying cardiovascular comorbidities.

Our study has several limitations. First, reliance on ICD-10 coding for HF diagnoses and HF events may reduce diagnostic accuracy and limit insight into HF severity and progression. In addition, we might underestimate the HF event if patients did not seek medical attention within the same healthcare system. Second, CDI episode details were unavailable, precluding analysis of how infection burden might influence outcomes, an important consideration given the increased CDI mortality in patients with HF [13], and its potential to exacerbate HF. Finally, our analysis was limited to patients who received bezlotoxumab, and we did not include a non-bezlotoxumab comparator group. As such, we cannot determine whether the higher rates of HF events reflect a direct effect of bezlotoxumab or the underlying cardiovascular risk of this population. On the other hand, it is challenging to redesign the current study to include a non-bezlotoxumab control group by solely using ICD-10 code A04.71 without chart review. Our goal was to describe real-world outcomes rather than establish causality, and we have avoided causal language in our interpretation. Future studies with matched comparators will be needed to clarify whether bezlotoxumab independently increases HF risk.

## 5. Conclusions

Using a large database analysis based on ICD codes, bezlotoxumab appears to be safe in patients without HF but is associated with an increased HF exacerbation risk in those with pre-existing HF, particularly HFrEF, or other cardiovascular comorbidities. Nevertheless, a control group (i.e., patients with CDI not treated with bezlotoxumab) was not included in the study. Although bezlotoxumab is currently unavailable on the market, future medications developed for CDI should include HF events in their safety evaluations and enroll patients with a history of HF in the study population.

## Figures and Tables

**Table 1:** Characteristics and factors of heart failure events in patients who received bezlotoxumab.

Characteristic	No (%)			Univariate Analysis		Multivariate Analysis ^c^	
Patients	Total	HF Event	No HF Event	Unadjusted HR (95% CI)	*p* Value	Adjusted HR (95% CI)	*p* Value
	N = 2515	N = 147	N = 2368				
**Age ≥ 65 y**	1586 (63)	119 (81)	1467 (62)	2.610 (1.715–3.972)	**<0.001**	-^b^	-^b^
**Sex**					0.401		
Male	1012 (40)	64 (44)	948 (40)	Reference			
Female	1503 (60)	83 (56)	1420 (60)	0.866 (0.619–1.212)			
**Race**					**0.005**	-^b^	-^b^
White	2014 (80)	117 (80)	1897 (80)	Reference			
Black	180 (7)	20 (14)	160 (7)	2.027 (1.228–3.345)			
Asian	40 (2)	1 (0.7)	39 (2)	0.416 (0.057–3.053)			
Others	281 (11)	9 (6)	272 (11)	0.537 (0.269–1.069)			
**History of HF**					**<0.001**		**<0.001**
No	2244 (89)	30 (20)	2214 (93)	Reference			
HFpEF	103 (4)	30 (20)	73 (3)	30.323 (17.370–52.936)		8.632 (4.497–16.568)	
HFrEF	168 (7)	87 (59)	81 (3)	79.252 (49.510–126.863)		19.400 (10.757–34.988)	
**Comorbidities**							
Atrial fibrillation	47 (2)	19 (13)	28 (1)	12.405 (6.746–22.811)	**<0.001**	-^b^	-^b^
Alcohol use disorder	99 (4)	13 (9)	86 (4)	2.574 (1.401–4.732)	**0.002**	-^b^	-^b^
Cancer	556 (22)	97 (66)	459 (19)	8.069 (5.651–11.520)	**<0.001**	-^b^	-^b^
CKD stage ≥ 3	301 (12)	88 (60)	213 (9)	15.090 (10.543–21.598)	**<0.001**	1.715 (1.029–2.857)	**0.039**
Diabetes mellitus	296 (12)	86 (59)	210 (9)	14.488 (10.134–20.711)	**<0.001**	-^b^	-^b^
HIV	10 (0.3)	5 (3)	5 (0.2)	16.641 (4.762–58.146)	**<0.001**	-^b^	-^b^
Hyperlipidemia	565 (22)	121 (82)	444 (19)	20.164 (13.038–31.185)	**<0.001**	3.184 (1.793–5.655)	**<0.001**
Hypertension	639 (25)	128 (87)	511 (22)	24.482 (14.973–40.031)	**<0.001**	-^b^	-^b^
Heart transplant	14 (0.5)	8 (5)	6 (0.3)	22.656 (7.754–66.197)	**<0.001**	7.485 (1.734–32.306)	**0.007**
Inflammatory bowel disease	147 (6)	15 (10)	132 (6)	1.925 (1.097–3.377)	**0.022**	-^b^	-^b^
Ischemic heart disease	372 (15)	108 (73)	264 (11)	22.070 (14.974–32.528)	**<0.001**	1.987 (1.128–3.502)	**0.018**
Non-HTx	106 (4)	20 (14)	86 (4)	4.179 (2.489–7.017)	**<0.001**	0.333 (0.161–0.688)	**0.003**
Obesity ^a^	531 (21)	48 (33)	483 (20)	1.892 (1.322–2.709)	**<0.001**	-^b^	-^b^
Obstructive sleep apnea	212 (8)	49 (33)	163 (7)	6.764 (4.635–9.871)	**<0.001**	-^b^	-^b^
Tobacco use	97 (4)	24 (16)	73 (3)	6.134 (3.737–10.069)	**<0.001**	-^b^	-^b^
Valvular heart disease	257 (10)	88 (60)	169 (7)	19.407 (13.472–27.957)	**<0.001**	2.267 (1.373–3.742)	**0.001**
**Medication**							
ACEI	110 (4)	13 (9)	97 (4)	2.272 (1.242–4.159)	**0.008**	-^b^	-^b^
ARB	91 (4)	13 (9)	78 (3)	2.848 (1.544–5.255)	**<0.001**	-^b^	-^b^
ARNI	6 (0.2)	3 (2)	3 (0.1)	16.424 (3.286–82.092)	**<0.001**	-^b^	-^b^
Beta-blocker	188 (7)	25 (17)	163 (7)	2.772 (1.752–4.386)	**<0.001**	-^b^	-^b^

Abbreviations: ACEI, angiotensin-converting enzyme inhibitor; ARB, angiotensin II receptor blocker; ARNI, angiotensin receptor-neprilysin inhibitor; CI, confidence interval; CKD, chronic kidney disease; HF, heart failure; HFpEF, heart failure with preserved ejection fraction; HFrEF, heart failure with reduced ejection fraction; HIV, human immunodeficiency virus; HR, hazard ratio; Non-HTx, other solid organ transplant except heart. ^a^ Defined as BMI ≥ 30 kg/m^2^. ^b^ Variables were entered into the initial Cox regression model based on *p* ≤ 0.25 in the univariate analysis and later removed from the final model owing to a lack of statistical significance (*p* ≤ 0.05) in the model through a backward elimination procedure. ^c^ The overall model test indicated statistical significance (χ^2^ = 509, *p* < 0.001). Fit statistics were as follows: Deviance = 611, AIC = 627, and BIC = 674. Pseudo-R^2^ measures demonstrated moderate to strong explanatory power, with McFadden’s R^2^ = 0.454, Cox & Snell R^2^ = 0.183, and Nagelkerke R^2^ = 0.509.

**Table 2:** Studies reporting heart failure events in patients treated with bezlotoxumab.

Reference	Author, Published Year, Study Type	Bezlotoxumab Group	Control Group (Placebo or Standard of Care)
[1,12]	Package insert, 2016 Clinical trials	Frequency of HF exacerbation during 12-week study period: ➢ 2% (17/786) of all study participants ➢ 13% (15/118) of patients with Hx of HF. 12-week mortality in patients with Hx of HF: 19% (23/118)	Frequency of HF exacerbation during 12-week study period: ➢ 0.9% (7/781) of all study participants ➢ 5% (5/104) of patients with Hx of HF. 12-week mortality in patients with Hx of HF: 13% (13/104)
[6]	Oksi et al., 2019, Retrospective multicenter	90-day HF exacerbation: 0/46 (0%) One patient who had end-stage coronary artery disease and congestive heart failure died 5 days after bezlotoxumab infusion. The treating physician regarded the patient’s demise as a natural event due to underlying comorbidities.	NA
[7]	Escudero-Sánchez et al., 2020, Retrospective multicenter	90-day HF exacerbation: 1/91 (1%). HF exacerbation occurred 86 days after bezlotoxumab infusion.	NA
[8]	Hengel et al., 2020, Retrospective multicenter	90-day HF exacerbation: 0/200 (0%). This study included 10 patients with Hx of HF, and 2 of them died. ➢ The first patient is an 80-year-old female with a Hx of interstitial lung disease, rheumatoid arthritis and HF. She developed respiratory failure and healthcare-associated pneumonia 1 week after bezlotoxumab infusion. She received bezlotoxumab following her 5th CDI episode and died 75 days after the infusion. ➢ The second patient is a 31-year-old female with systemic lupus erythematosus, end-stage renal disease, HF, and myocarditis status post-mitral valve replacement. She developed generalized weakness with lower-extremity edema 6 days after bezlotoxumab infusion, which was attributed to nonfunctional peritoneal dialysis. She received bezlotoxumab following her 6th CDI episode and died 40 days after the infusion.	NA
[9]	Johnson et al., 2022 Matched retrospective single center	90-day HF exacerbation: 1/7 (14%) in patients with Hx of HF. HF exacerbation occurred 4 weeks after bezlotoxumab infusion.	90-day HF exacerbation: 1/9 (11%) in patients with Hx of HF.
[10] (conference abstract)	Bradley et al., 2022, Retrospective single center	90-day HF exacerbation: 1/10 (10%) HF exacerbation occurred 69 days after bezlotoxumab infusion.	NA
[11] (conference abstract)	Chaar et al., 2023, Retrospective single health system	30-day and 90-day HF exacerbation: ➢ Hx of HFpEF: 0/25 (0%); 0/25 (0%) ➢ Hx of HFrEF: 2/16 (13%), 2/15 (13%) ➢ 30-day and 90-day all-cause mortality ➢ Hx of HFpEF: 0/25 (0%); 0/25 (0%) ➢ Hx of HFrEF: 1/16 (6%), 1/15 (6%)	NA
Present study	Chiu et al., 2026, Large database analysis	90-day new onset heart failure or HF exacerbation: ➢ Overall: 6% ➢ No Hx of HF: 1% ➢ Hx of HFpEF: 29% ➢ Hx of HFrEF: 52% 90-day all-cause mortality: ➢ Overall: 0.9% ➢ No Hx of HF: 0.04% ➢ Hx of HFpEF: 4% ➢ Hx of HFrEF: 11%	NA

Abbreviations: HFpEF, heart failure with preserved ejection fraction; HFrEF, heart failure with reduced ejection fraction; Hx: history; NA, not applicable.

## Data Availability

The de-identified datasets generated during and/or analyzed during the current study are available from the corresponding author on reasonable request.

## Supplementary Materials

The following supporting information can be downloaded at: https://www.mdpi.com/article/10.3390/idr18020028/s1, Supplementary Methods; Table S1: Diagnosis codes used in this study; Table S2: Logical Observation Identifiers Names and Codes (LOINC) for laboratory values used in this study; Table S3: RxNorm codes used in this study; Figure S1: Schematic representation of the study design.
