# Supplementary material for: Heart Failure Incidence and Risk Factors in U.S. Adults Receiving Bezlotoxumab: A Large Database Analysis
Source: Infect Dis Rep. 2026 Mar 31;18(2):28. doi: 10.3390/idr18020028 (PMC13116173; doi:10.3390/idr18020028)

Supplementary Material

Supplementary Methods

Supplementary Table S1. Diagnosis codes used in this study

Supplementary Table S2. Logical Observation Identifiers Names and Codes (LOINC) for laboratory values used in this study.

Supplementary Table S3. RxNorm codes used in this study.

Supplementary Figure S1. Schematic representation of the study design.

Supplementary Methods

Global Federated Research Network

TriNetX aggregates anonymized data from approximately 100 million patients of all ages across more than 100 medical centers in the US, Canada, Europe, Australia, Indonesia, and other countries. Each participating healthcare organizations (HCOs) contribute data from electronic medical record (EMR) systems used for patient care. Received data are either structured (e.g., coded diagnoses) or unstructured (e.g., provider notes) and processed by Natural Language Processing (NLP) Technology. Most participating HCOs are large academic medical institutions with both inpatient and outpatient facilities. The data provided represents the entire patient population at each HCO. Most give an average of eight years of historical data.

TriNetX receives data directly from each HCO's research repository in the TriNetX environment, or the HCO sends comma-separated value (CSV) files coded in the TriNetX Data Dictionary. Data providers update their information regularly, with over 80 percent of participants refreshing every one-, two-, or four weeks. The average lag time for an HCO's source data to refresh is one month.

TriNetX maps the data to a standard, controlled set of clinical terminologies and enters clinical information into a proprietary data model. The model entry includes extensive data quality assessment, and any records failing to meet the TriNetX quality standards are rejected. TriNetX is certified by the International Organization for Standardization (ISO) 27001:2013 and maintains an Information Security Management System (ISMS) to protect patient healthcare data and satisfies the requirements of the HIPAA Security Rule. Individualized patient data were obtained directly from the TriNetX platform in several CSV files. For patients with multiple encounters, we ensured the appropriate counting of distinct events.

Missing data

Data for the following variables were missing and were excluded from further analysis: date of birth, gender, race, and BMI.

Ethics Statement

Any data displayed on the TriNetX platform in aggregate form, or any patient-level data provided in a data set generated by the TriNetX platform, only contains de-identified data as per the de-identification standard defined in Section 164.514(a)

of the Health Insurance Portability and Accountability Act (HIPAA) Privacy Rule. The process of de-identifying data is attested to through a formal determination by a qualified expert as defined in Section 164.514(b)(1) of the HIPAA Privacy Rule. Geographic reporting at the regional level prevents potential re-identification through the localization of patients or HCOs. Research utilizing TriNetX does not require ethical approval because patient-identifiable information is not accessible to users.

Supplementary Table S1. Diagnosis codes used in this study[a]

	**ICD-10-CM**
**Alcohol use disorder (AUD)**	F10
**Atrial fibrillation (Afib)**	I48
**Chronic kidney disease (CKD), ≥ stage 3**	N18.3, N18.4, N18.5
**Clostridium difficile infection**	A04.7
**Diabetes mellitus (DM), type 2**	E11
**Heart failure (HF)**	I50
**Heart failure exacerbation**	I50.21, I50.23, I50.31<br>I50.33, I50.41, I50.43
**Heart failure with preserved ejection fraction**	I50.3
**Heart failure with reduced ejection fraction**	I50.2
**Heart transplant (HTx)**	Z94.1
**Lung transplant**	Z94.2
**Kidney transplant**	Z94.0
**Liver transplant**	Z94.4
**Human immunodeficiency virus (HIV)**	B20
**Hyperlipidemia (HLD)**	E78
**Hypertension (HTN)**	I10, I11, I12, I13, I14, I15
**Inflammatory bowel disease (IBD)**	K50, K51
**Ischemic heart disease (IHD)**	I20, I21, I22, I23, I24, I25
**Malignancy**	C00 – D49
**Sleep apnea (OSA)**	G47.3
**Tobacco use**	Z72.0
**Valvular heart disease (VHD)**	I34, I35, I36, I37

a. Comorbidities and medications were assessed within 1 year prior to bezlotoxumab infusion.

Supplementary Table S2. Logical Observation Identifiers Names and Codes (LOINC) for laboratory values used in this study.

	**LOINC**
**Body Mass Index (BMI)**	39156-5

Supplementary Table S3. RxNorm codes used in this study. [a]

	RxNorm
**Bezlotoxumab**	1855048
**ACEI**	captopril (1998), enalapril (3827), lisinopril (29046), ramipril (35296)
**ARB**	candesartan (214354), losartan (52175), valsartan (69749)
**ARNI**	sacubitril/valsartan (1656328)
**Beta-blocker**	bisoprolol (19484), carvedilol (20352), metoprolol (6918)

Abbreviation: ACEI, Angiotensin-converting enzyme inhibitors; ARB, Angiotensin II receptor blockers; ARNI, Angiotensin receptor neprilysin inhibitor; HF, heart failure.

a. Comorbidities and medications were assessed within 1 year prior to bezlotoxumab infusion.

Supplementary Figure S1. Schematic representation of the study design.

Abbreviations: HFpEF, heart failure with preserved ejection fraction; HFrEF, heart failure with reduced ejection fraction; Hx: history.

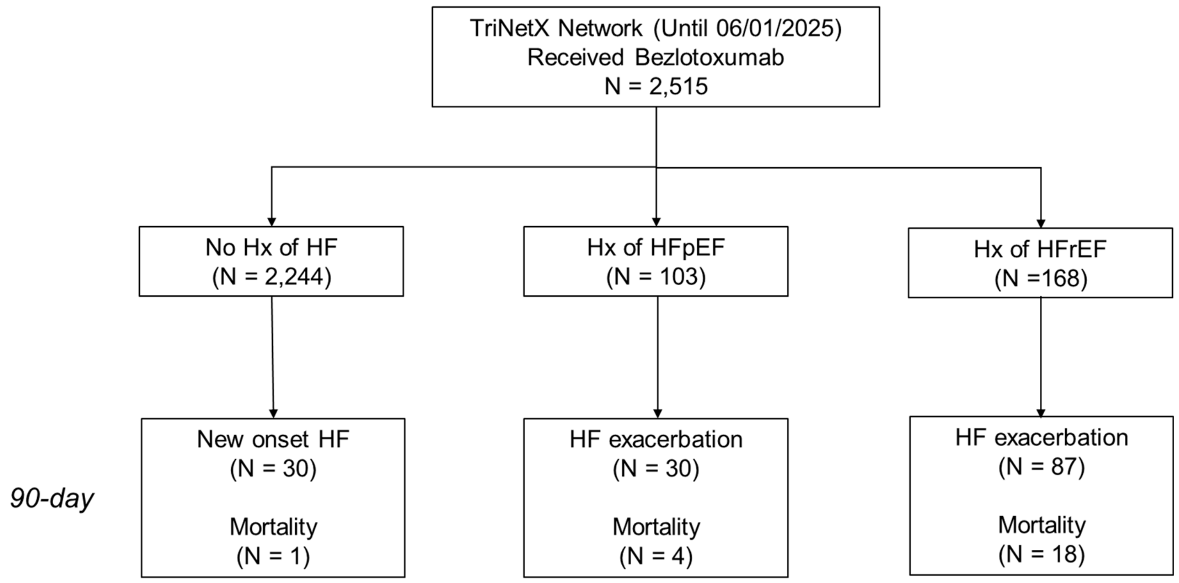

Supplement: Supplementary file 1 [file idr-18-00028-s001.zip › idr-4184728-supplementary.pdf]
